# Characterization of Evolutionarily Conserved* Trypanosoma cruzi* NatC and NatA-N-Terminal Acetyltransferase Complexes

**DOI:** 10.1155/2019/6594212

**Published:** 2019-03-06

**Authors:** Stephen Ochaya, Oscar Franzén, Doreen Asiimwe Buhwa, Håvard Foyn, Claire E. Butler, Svein Isungset Stove, Kevin M. Tyler, Thomas Arnesen, Enock Matovu, Lena Åslund, Björn Andersson

**Affiliations:** ^1^Department of Cell and Molecular Biology, Karolinska Institutet, Box 285, 171 77 Stockholm, Sweden; ^2^Department of Immunology and Microbiology, Gulu University, P.O. Box, 166 Gulu, Uganda; ^3^Department of Parasitology and Microbiology, Makerere University, P.O. Box 7062, Kampala, Uganda; ^4^Department of Biological Sciences, University of Bergen, N-5020 Bergen, Norway; ^5^Biomedical Research Centre, Norwich Medical School, University of East Anglia, Norwich, Norfolk, NR4 7TJ, UK; ^6^Department of Biomedicine, University of Bergen, 5020 Bergen, Norway; ^7^Department of Surgery, Haukeland University Hospital, N-5020 Bergen, Norway; ^8^Department of Immunology, Genetics and Pathology, Rudbeck Laboratory, 75185 Uppsala, Sweden

## Abstract

Protein N-terminal acetylation is a co- and posttranslational modification, conserved among eukaryotes. It determines the functional fate of many proteins including their stability, complex formation, and subcellular localization. N-terminal acetyltransferases (NATs) transfer an acetyl group to the N-termini of proteins, and the major NATs in yeast and humans are NatA, NatB, and NatC. In this study, we characterized the* Trypanosoma cruzi* (*T. cruzi)* NatC and NatA protein complexes, each consisting of one catalytic subunit and predicted auxiliary subunits. The proteins were found to be expressed in the three main life cycle stages of the parasite, formed stable complexes* in vivo*, and partially cosedimented with the ribosome in agreement with a cotranslational function. An* in vitro* acetylation assay clearly demonstrated that the acetylated substrates of the NatC catalytic subunit from* T. cruzi* were similar to those of yeast and human NatC, suggesting evolutionary conservation of function. An RNAi knockdown of the* Trypanosoma brucei* (*T. brucei*) NatC catalytic subunit indicated that reduced NatC-mediated N-terminal acetylation of target proteins reduces parasite growth.

## 1. Introduction

Trypanosomes are protozoan parasites that can cause severe health problems, mainly in developing countries.* Trypanosoma cruzi* is the causative agent of Chagas disease, common throughout Latin America, while* T. brucei*, mainly present in Africa, causes sleeping sickness in humans and Nagana in livestock [1, 2]. There is no vaccine against trypanosome-related diseases and the available drugs cause serious side effects [3, 4]. The study about N-terminal acetylation as a possible chemotherapeutic target to fight parasite infections is limited. Protein N*α*-acetylation (Nt-acetylation) is an irreversible protein modification where the acetyl moiety is transferred to the N*α* amino group of a protein or polypeptide by N-terminal acetyltransferases (NATs). NATs are grouped according to their substrate specificity. In humans, seven NATs have been identified so far (NatA-F and NatH) [5, 6]. Of these, NatA, NatB, and NatC have the largest number of substrates and have been characterized extensively. The human NatA protein complex is composed of a catalytic subunit (hNaa10) and an auxiliary subunit (hNaa15) and the human NatC consists of a catalytic (hNaa30) subunit and two auxiliary (hNaa35 and hNaa38) subunits [7, 8]. The proteins form stable complexes* in vivo* and cosediment with the ribosome [8, 9]. Of late, studies exploring the biological significance of NATs have become topical, in particular with regard to how they contribute to cellular integrity and their roles in cancer [10, 11]. At the substrate protein level, Nt-acetylation may act as a degradation signal [12], mediate protein complex formation [13], or inhibit post-translational ER-translocation [14]. Indeed, both human NatC and NatA have been suggested as possible target to control cancer [8, 15].

In N-terminal acetyltransferases (NATs), the co- and posttranslational modification is common in all kingdoms of life. About 60 %, 90 %, 75 %, and 18 % of yeast, human, plant, and archaea proteins, respectively, are thought to be Nt-acetylated [5, 16, 17].

The NatA complex from* T. brucei* has been found to be essential for cell viability in both the mammalian and insect stages [18]. We previously characterized a novel acetyltransferase, the catalytic subunit of the NatC complex in* T. cruzi* [19], thought to belong to the NatC subgroup. In the present study, we have characterized and begun to investigate the biological significance of the predicted NatC and NatA in* T. cruzi*.

We demonstrate that the catalytic subunits (TcNaa10 and TcNaa30), and the predicted auxiliary subunits are expressed and cosediment with the ribosome. We find that TcNaa30 catalyzes the acetylation of N-termini similar to those acetylated by NatC in yeast (yNaa30) and hNaa30* in vitro* and our analyses indicate that the protein may function both as a N*α*- and as a N*ε*-acetyltransferase. Finally, there is an indication that the knockdown of the* T. brucei* NatC catalytic subunit is important to the parasite.

## 2. Materials and Methods

### 2.1. Cell Culture

*T. cruzi* CL Brener epimastigotes were cultured as previously described [19]. Tissue culture derived trypomastigotes were obtained through infection of a Vero cell monolayer, harvesting the media by centrifugation at 1640 g for 10 min. Amastigotes were obtained by harvesting 5 x 10^6^ trypomastigotes per ml and incubating in serum-free DMEM for 48 h at 37°C. Metacyclogenesis was induced by separating 5 x 10^6^ epimastigotes per ml into Grace's insect medium supplemented by intestinal homogenate from* Rhodnius prolixus* [20] and 0.5 % pyruvate-glutamate-antibiotic (PGAB).* T. brucei* strains were grown in HM-I9 medium at 37°C, 5 % CO_2_.

### 2.2. Identification, Cloning, and Expression of Suspected TcNatC and TcNatA Subunits

Human Mak3 (hNaa30), gene ID 122830, and hNaa38 (NP_001317040.1 GI:1052793474) sequences were BLASTed against the* T. cruzi* CL Brener proteome in order to identify* T. cruzi* homologs of NatC catalytic and an auxiliary subunit. Similarly, the second TcNatC auxiliary subunit was identified using plant Mak10 acetyltransferase* Arabidopsis thaliana* NP_001118295.1, GI:186500070 or* Rattus norvegicus* NP_579858.1 GI:19033372. The auxiliary subunits were amplified from total genomic DNA from Sylvio strain (TcI) using the following primers TcNATC mak10 Forward: 5′ C**GAATTC**ATGGCGTGTGACCTTGA 3′, and TcNATC mak10 Reverse: 5′ GA**GCGGCCGC**TTACCTGGCTTCCTTCTTG 3′, with EcoR1 and Not1 restriction sites (underlined), respectively, and, TcLsmd1 Forward: 5′ G**GAATTC**ATGGGCCGCGAGAGCATGCTTCACAA 3′ and TcLsmd1 Reverse: 5′AAG**CTCGAG**TTAGCGCTTCCGCTT 3′, with EcoR1 and XhoI restriction sites (underlined). The genes were cloned into pGEX5-1 vector expressing GST (glutathione S-transferase) and recombinant proteins were produced. The pelleted bacteria was dissolved in PBS containing EDTA-free protease inhibitor tablets (Roche), 1 mM EDTA, and 100 *μ*g lysozyme and incubated on ice for about 15 min. Sarkosyl was added to a concentration of 1.5 %. The cells were briefly sonicated four times, 10 s each, with 30 s pauses using Branson sonifier cell disruptor B 15. The pellet and supernatant were analyzed to detect the presence of the induced protein.

For the TcNatA subunits, human or yeast NatA sequences were used as queries in the NCBI BLAST database in order to identify TcNatA homologues.* T. cruzi* gene TcCLB.506227.230 (predicted catalytic subunit, which we named TcNaa10) and gene TcCLB.510301.80 (predicted auxiliary subunit, named TcNaa15) were identified. The genes were amplified from genomic DNA using the following primers: TcNAA10 Forward: 5′ AA**GAATTC**ATGCAGATCCGTCGC 3′, TcNAA10 Reverse: 5′AAA**CTCGAG**TCACTTTTTCGTCTTGCC 3′, TcNAA15 Forward: 5′ATCG**GAATTC**CGGTAGTGCTTCCTCCGGCG 3′, and TcNAA15 Reverse: 5′ATCG**CTCGAG**GCGCTGGCCAACACCTCATCA 3′. Bold and underlined are EcoRI (forward) and XhoI (reverse) restriction sites.

The genes were subsequently cloned into the pGEX5-1 vector expressing GST (glutathione S-transferase) yielding pGEX5-1-TcNaa10 and pGEX5-1-TcNaa15. The reading frames were confirmed as described previously [19]. Bacterial Top10 cells (Invitrogen) transformed with pGEX5-1-TcNaa10 were grown at 37°C until approximately OD_600_ 0.5 and induced with 0.3 mM isopropyl *β*-D-1-thiogalactopyranoside (IPTG). Cells were grown and processed as described before [19], except that protease inhibitor (EDTA-free tablet inhibitor from Roche) was used. The bacterial Top10 cells (Invitrogen) transformed with pGEX5-1-TcNaa15 were grown to approximately OD_600_ 0.8 and induced with 0.1 mM IPTG at 24°C for about 24 h and further processed as was done for pGEX5-1-TcNaa10.

### 2.3. Generation of Anti-TcNaa10, TcNaa15, and Anti-TcNaa38 Antibodies and Western Blot Analysis

The antibody production for TcNaa10 and TcNaa38 was performed in a rabbit by Innovagen (Lund, Sweden), as before [19]. Protein G was used to purify the IgGs. The anti-GST antibodies were removed by passing the immunoglobulin through a GST-column. The depletion and titer were evaluated by immunoblotting to GST and GST-TcNaa38 and GST-TcNaa10 electrotransferred strips (not shown). Antibody against TcNaa15 was generated by the Agrisera company (Umeå-Sweden) by inoculating one rabbit with synthetic peptides: Naa15-EL (700-712) (NH2-) CDEVLASAWEKIKE (-COO). The peptide sequence was selected from conserved regions from both CL Brener haplotypes (non-Esmeraldo and Esmeraldo like). Western blotting was performed using standard procedures and as before [19]. The anti-TcNaa10, anti-TcNaa15, and anti-TcNaa38 antibodies were used at a dilution of 1:4000, 1:2000, and 1:2000, respectively. For comparison between life cycle stages, fractions containing 10^6^ cells were lysed directly in sample loading buffer and separated on a 15 % acrylamide gel. Proteins were transferred using a semidry system and the membrane probed with, for example, anti-TcNaa38 overnight.

### 2.4. In Vitro Acetylation Assay

*E. coli* cells harboring the expression plasmid pGEX5-TcNaa30 were grown at 37°C in Luria-Bertani medium containing appropriate amounts of ampicillin. Expression was induced at approximately 0.5 OD600 by the addition of 0.3 mM IPTG and growth was continued for additional 18 h at 17°C, 190 rpm. The cells were processed as described previously [19].

The enzyme activity of purified GST-TcNaa30 was determined as described in [8]. In brief, GST-TcNaa30 was mixed with potential oligopeptide substrates (300 mM) and acetyl-CoA (300 mM) in a total volume of 60 *μ*l acetylation buffers. The samples were incubated at 37°C for 30 min. The enzyme activity was quenched by adding 5 *μ*l of 10 % TFA. The amount of acetylated oligopeptides was determined based on the absorbance at 215 nm after analysis with RP-HPLC. Synthetic Peptide Sequences used were as described elsewhere [7, 8].

To assess the TcNaa10 acetyltransferase activity, recombinant protein was expressed as described above, except that growth was continued for another 25 h at 17°C, 189 rpm. The cells were chilled on ice and harvested by centrifugation at 5000 rpm for 15 min. The cell pellet was suspended in 5 ml of ice-cold PBS containing EDTA-free tablets inhibitor (Roche). Cells were sonicated 4 times, 10 s each using a Branson sonifier cell disruptor B 15. Five ml of cold PBS + inhibitor, 0.5 ml of 20 % Triton X-100 (final conc. 1 %) was added and the cells were incubated for 30 min at 4°C and thereafter centrifuged for 15 min at 10,000 rpm. From a 50 % slurry of glutathione-Sepharose 4B (GE Healthcare), about 250 *μ*l was added to the supernatant and the mixture was incubated for 2-3 h at 4°C. The beads were washed three times with cold PBS containing 1 % Triton X-100, followed by one wash with PBS. The amount of protein on the beads was estimated from Coomassie staining of SDS-PAGE gels.

The purified recombinant protein (GST-TcNaa10), eluted from the beads, was incubated with acetyl-CoA and synthetic peptides suggested to be the substrates for NatA. The activity of the enzyme was stopped after 30 min and the results analyzed by HPLC.

### 2.5. Immunofluorescence Microscopy

Parasites were prepared for IF essentially as previously described [21], fixing in 4 % paraformaldehyde for 5 min. Cells were then blocked in 10 % goat serum and primary antibodies were used at a 1:50 dilution for 1 h. Anti-rabbit AlexaFluor488 was used to recognize the primary antibodies and cells were DAPI stained prior to mounting in Fluoromount. Imaging was achieved using a Zeiss Axioplan2 microscope and Axiovision 4.7 software.

### 2.6. Immunoprecipitation (IP)

Approximately 10^9^ parasites per ml were used for immunoprecipitation. Exponentially growing cells were lysed in lysis buffer [0.75 % CHAPS detergent, 1 mM MgCl2, 1 mM EGTA, 5 mM *β*- mercaptoethanol, 10 mM Tris-HCL (pH 7.6), 10 % glycerol, and 1 mM Pefabloc (Roche)]. The sample was incubated on ice and later centrifuged. The supernatant was precleared by incubation with protein A/G–agarose (Santa Cruz Biotechnology) on a roller for 1 h at 4°C. The beads were removed by centrifugation at 1000 g for 3 min. The cell lysate was incubated with about 2 *μ*g of anti-TcNaa10 or anti-TcNaa30 antibody. As a control, one part of the lysate was incubated with rabbit sera (preimmune). Both samples were incubated on a roller for about 2.5 h at 4°C before adding 30 *μ*l of Protein A/G–agarose beads and further incubated overnight at the same temperature. The beads were collected by centrifugation as above, washed, mixed with sample buffer, and boiled for 10 min. After centrifugation, the supernatant was analyzed by SDS/PAGE and western blotting. Reciprocal IP with anti-TcNaa15 or anti-TcNaa38 was done as described except that, in this case, the lysate was not precleared.

### 2.7. Polysome Isolation

Total ribosome isolation was performed using a modification of previously described methods [8]. Approximately 10^9^ cells were used per experiment. Prior to harvesting, parasites were treated with 100 *μ*g/ml cycloheximide (CHX) for about 10 min on ice. Cells were then lysed with KCl ribosome lysis buffer [8] and incubated on ice for 15 min. Cells were homogenized by repeated pipetting and the homogenate verified with light microscopy. The lysate was centrifuged at 18000 g at 4°C for 5 min using Beckman rotor 25.50. One ml of the lysate was overlaid on 3 ml of 25 % sucrose cushion sucrose and ultracentrifuged at 135,715.5 g for 2 h using Sorvall AH-650 rotor (Beckman). The pellet was dissolved in ribosomal lysis buffer. Total parasite lysate, top supernatant (post-polysome lysate), and ribosomal pellet were analyzed by SDS-PAGE and western blotting.

### 2.8. Nuclear and Cytoplasmic Preparation

About 10^7^ exponentially growing parasites were washed twice in PBS and lysed in 10 ul of TELT buffer (50 M Tris-HCL pH 8, 62.5 mM EDTA, 2.5 M LiCl, 0.4 % Triton X-100, and 100 mg/ml lysozyme). Thereafter, NE-PER Nuclear and Cytoplasmic Extraction Reagents kit from Thermo Scientific was used according to recommendation, but with double amount of reagents. Prior to the use of the kit, the parasites were lysed by TELT, as the detergent provided with the kit did not lyse the parasite at the condition tested. Anticyclophilin A (kindly provided by Jacqueline Búa, Instituto Nacionale de Parasitologia, Buenos Aires, Argentina) and antihistone 3 from Upstate (Millipore) were used as positive control for cytoplasmic and nuclear proteins, respectively.

### 2.9. Bioinformatics

Homology searches were performed using the NCBI BLAST server. Extracted protein sequences were aligned using Clustal Omega multiple sequence alignment tool. ScanProsite and InterPro Protein sequence analysis and classification tools were used to identify domains.

### 2.10. Generation of Predicted T. brucei NatC Catalytic Subunit (TbNaa30) RNAi Cell Lines

The putative protein-coding region comprising nucleotides (216-788) of the predicted TbNaa30 (Tb927.7.2360), that is, a 573 bp fragment, was PCR-amplified using forward primer TbNatC- Naa30RNAi: 5′ATCG**GGATCC**CTACGGATGTCGCTCCTAGC 3′ and reverse primer TbNatC- Naa30RNAi: 5′ATCG**AAGCTT**GTAGCGCGGCAGAAATTTAG 3′. Underlined are BamHI and Hind III restriction sites, respectively. The PCR product was subcloned into tetracycline-inducible RNAi vector p2T7-177 using the restriction sites to yield p2T7-TbNaa30. The presence of the insert was verified by digesting the plasmid with respective enzymes. For easy incorporation into the chromosome, resulting plasmid (about 10 *μ*g) was linearized with NotI and transfected into* T. brucei brucei* 427 strain by electroporation, using about 2 x 10^7^ cells. Nonlinearized plasmid and mock transfection were used as negative controls. The transformants were selected with phleomycin (2.5 *μ*g). To confirm if the transfection was successful, DNA was extracted from the surviving parasites and PCR was performed to amplify the phleomycin gene fragment (350 bp) using specific primers (Phleo Forward 5′ ATG GCC AAG TTG ACC AGT GCC 3′ and Phleo Reverse 5′ TGC ACG CAG TTG CCG GCC GGG 3′). The starting parasite density of 2.5 x 10^4^ /ml was used and RNAi was induced using 100 ng of tetracycline. The same parasite density was used for the transformants and wild type (*T. b. brucei* 427). The noninduced/wild type and induced cells were examined and counted daily using light microscopy. Samples for gene/protein expression analyses were harvested daily for five days.

## 3. Results

### 3.1. Identification and Sequence Analysis of TcNat Proteins

The catalytic subunit of the TcNatC complex was identified by blast analysis and as previously described [19] (Table 1). In accordance with the recommended nomenclature [22], we now refer to this gene as TcNaa30. Similarly, we identified putative genes for the* T. cruzi* homologues of NatC auxiliary subunits (TcNaa35 and TcNaa38) (Table 1). The gene showed 19 %, 22 %, 49 %, and 62 % sequence identity at the aa level to its rat, plant,* Leishmania major,* and* T. brucei* counterpart, respectively. For TcNaa38, gene Tc00.1047053507209.10 (Tc9.10) was identified as the likely TcNatC subunit (Table 1). At the aa level, the TcNaa38 gene shares 32 %, 38 %, 57 %, and 62 % sequence identity with its yeast, human,* Leishmania major,* and* T. brucei* counterpart, respectively.

*T. cruzi NatA* homologs were identified by comparing with human Naa10 (gene ID 728880) (Table 1). The* T. cruzi* gene TcCLB.506227.230 (Tc7.230) was found to share 60 % and 42 % identity at the amino acid (aa) level with the human and yeast genes, respectively. We now refer to this gene as TcNaa10 according to the latest nomenclature [22]. In a search to identify the* T. cruzi* NatA auxiliary subunit, we used hNaa15 (NP_476516.1), as a BLAST query sequence. As seen in Table 1, the search identified CL Brener gene TcCLB.504163.110 (Tc3.110) and TcCLB.510301.80 (Tc01.80), with 29 % and 28 % sequence identity at the aa level, respectively. Both alleles were annotated as putative N-acetyltransferase subunit Nat1 and named TcNaa15. Sequence comparison of predicted TcNaa35, TcNaa38, TcNaa10, and TcNaa15 with selected species is displayed in (Supplementary Figure S1).

### 3.2. Expression and Recombinant Production of TcNatA and TcNatC Protein Subunits

We previously expressed the putative TcNaa30 and showed that it has autoacetylation enzyme activity [19]. To further characterize the TcNatC and TcNatA protein complex, we cloned the TcNaa35 and TcNaa38 ORFs and produced recombinant protein (Supplementary Figures S2A and S2B show the recombinant protein (GST-TcNaa35 and GST-TcNaa38) with an expected size of about 110 and 40 kDa, respectively). The annotated proteins of TcNaa10 and TcNaa15 have predicted molecular weights of 29.4 and 82.9 kDa, respectively, and we again produced recombinant proteins. TcNaa10 was initially insoluble (Supplementary Figures S2C and S2D) and was dissolved in sarkosyl as described previously [23].

### 3.3. Expression of TcNaa38/TcNaa30 and TcNaa10/TcNaa15 in the Parasite

To investigate the expression pattern of TcNatC and TcNatA, we used western blot to detect the proteins in the different stages of the parasite life cycle. In the study, polyclonal antibodies were produced in rabbit against the whole protein. The antibody against TcNaa15 was generated in a rabbit using synthetic peptides. For all the proteins assessed, we first carried out western blot analysis for preimmune rabbit sera, and, as expected, no band/signal was detected (not shown).

**Figure 1:**
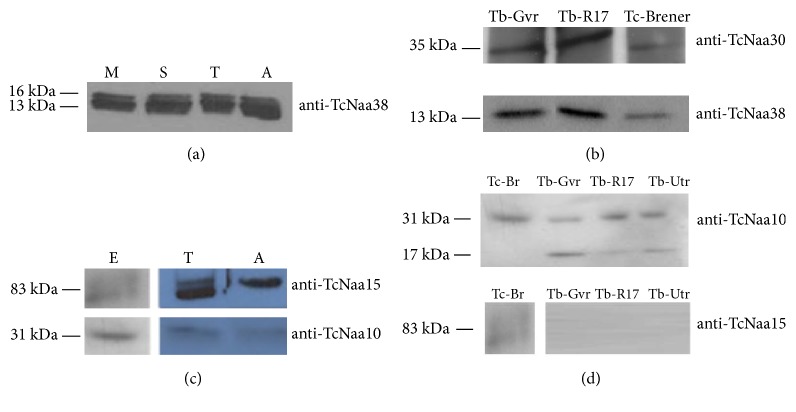
Expression of TcNaa38/TcNaa30 and TcNaa10/TcNaa15 in trypanosomes. (a) Total proteins from different stages of* T. cruzi* life cycle, epimastigote midlog (M), epimastigote stationary phase (S), trypomastigote (T), and amastigote (A) were used for western blotting. (b) Cross reaction of anti-TcNaa30 and anti-TcNaa38 against different* T. brucei* strains. Tb-Gvr (*T. brucei* GVR strain), Tb-R17 (*T. brucei* R17 strain), and Tc-Brener epimastigote (*T. cruzi* CL Brener strain). (c) Developmental stage expression of TcNaa10 and TcNaa15 in CL Brener strain epimastigote (E), trypomastigote (T), and amastigote (A). Purified anti-TcNaa10 and anti-TcNaa15 (1:4000, and 1: 2000 dilutions) were used for western blotting. (d) Cross reaction of anti-TcNaa10 (upper panel) and anti- TcNaa15 (lower panel) against different* T. brucei* strains. The* T. brucei* strains used were GVR35 (Gvr), R17 and UTRO 091B (Utr).

Analysis showed that TcNaa38 was expressed in the three main stages,* i.e*., in the epimastigote, trypomastigote, and amastigote stages of* T. cruzi* CL Brener (Figure 1(a)). However, multiple bands of similar size were recognized in all the stages, possibly due to posttranslational processing of proteins. The identity of the extra bands has not been investigated in this study. Anti-TcNaa38 and anti-TcNaa30 also recognized, for example, GVR35 [24] and UTRO [25] strains of* T. brucei* proteins (Figure 1(b)). TcNaa10/TcNaa15 were also found to be expressed in epimastigote, trypomastigote, and amastigote stages of the* T. cruzi* CL Brener strain (Figure 1(c)). Anti-TcNaa15 detected additional bands in all developmental stages, except amastigotes. The identities of these bands are not known. Furthermore, the result suggests an upregulation of TcNaa15 in the trypomastigote and amastigote stages with an opposite effect seen for TcNaa10, that is, downregulated in trypomastigotes and amastigotes (Figure 1(c)). As displayed in (Figure 1(d)), anti-TcNaa10 was found to cross react with* T. brucei*, while anti-TcNaa15, as expected, did not. In contrast to* T. cruz*i, anti-TcNaa10 recognized an extra band of 17 kDa in* T. brucei*, with no known identity. Taken together, the results indicate that the TcNatA and TcNatC protein complexes are constitutively expressed in* T. cruzi*.

### 3.4. Localization of TcNaa30 and TcNaa10/TcNaa15 by Fractionation

**Figure 2:**
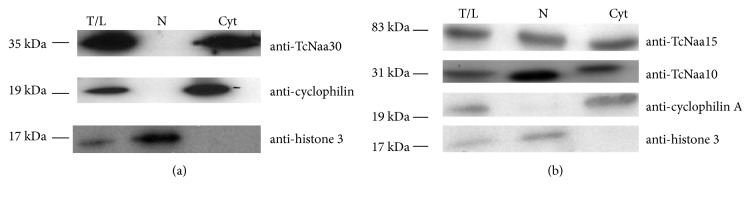
Localisation of TcNaa30 and TcNaa10/TcNaa15 in* T. cruzi*. (a) TcNaa30 localization in epimastigotes by western blotting. (b) Localization of TcNaa10/TcNaa15. Cytoplasmic (Cyt) and nuclear (N) fractions were assessed for the presence of TcNaa30, TcNaa10, and TcNaa15. Anticyclophilin A and antihistone 3 were used as positive controls for cytoplasmic and nuclear protein, respectively. T/L indicates total cell lysate.

As shown in [19], the staining profile of the putative TcNaa30 was predominantly located in the cytoplasm, and we now observed the same result by fractionation (Figure 2(a)). For TcNaa15 and TcNaa10, both proteins showed nuclear and cytoplasmic location (Figure 2(b)).

### 3.5. Subcellular Localization of TcNaa30/TcNaa38 and TcNaa10/TcNaa15 by Immunofluorescence

**Figure 3:**
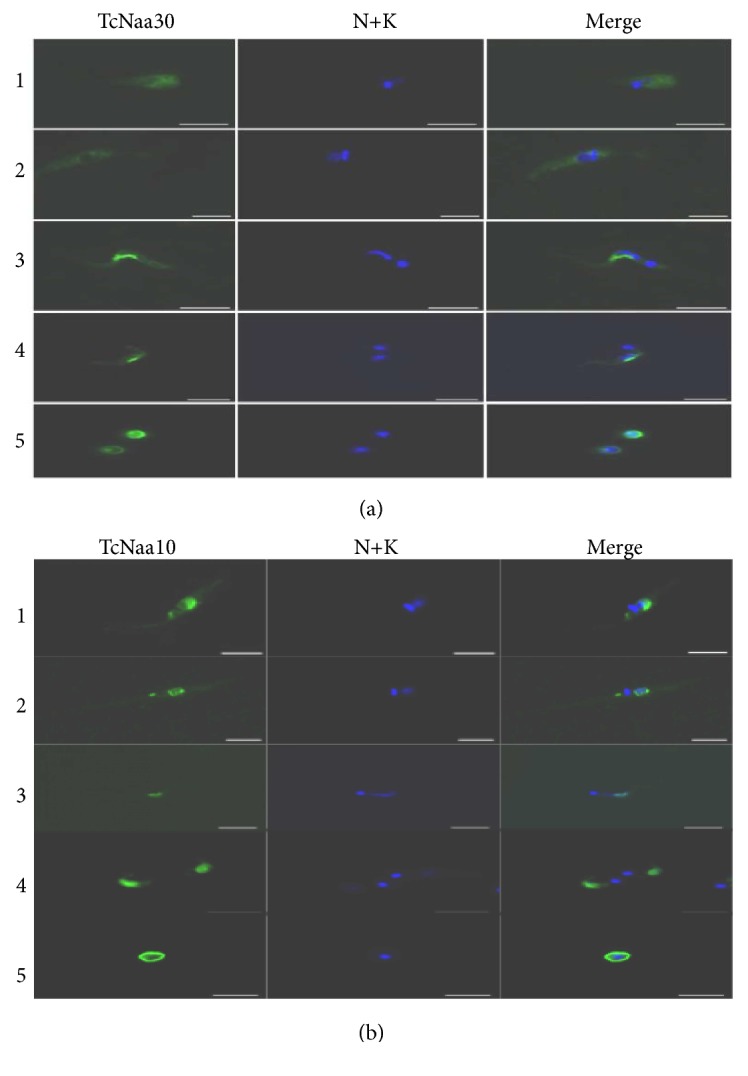
Localization of TcNaa30 and TcNaa10 by immunolabelling. Numbers 1 to 5 denotes midlog epimastigotes, Stationary epimastigotes, metacyclic trypomastigotes, Trypomastigotes, and amastigotes, respectively.* T. cruzi* four life cycle stages were immunolabelled with (a) anti-TcNaa30 and (b) anti-TcNaa10. The nucleus and kinetoplast were visualized using DAPI stain (N+K), scale bars = 5*μ*m.

In assessing the staining patterns of the four proteins, no staining was observed by preimmune sera or by secondary antibody alone (data not shown). In both midlog and stationary epimastigotes, the TcNaa30 exhibited some perinuclear accumulation and punctate structures, particularly in the stationary phase (Figure 3(a)). In both metacyclic and tissue culture derived trypomastigotes, TcNaa30 appeared to be relatively sequestered in a perinuclear distribution, as was observed in [26]. When trypomastigotes were differentiated into amastigotes* in vitro*, however, more peripheral staining was observed (Figure 3(a)). These differences could possibly be related to differential regulation of protein trafficking.

TcNaa38 staining was predominantly punctate and cytoplasmic labeling more diffuse at the midlog stages (Supplementary Figure S3A). Tissue culture derived trypomastigotes showed a diffuse cytoplasmic localization with some perinuclear accumulation. In amastigotes* in vitro*, TcNaa38 staining was again punctate and cytoplasmic (Supplementary Figure S3A). Using a Vero cell monolayer to assess intracellular amastigotes, the staining profile showed a diffuse localization of TcNaa30 in the cytoplasm, and a more punctuated labeling for TcNaa38 (Supplementary Figure S4).

The localization pattern of TcNaa10 and TcNaa15* in vivo* in the four developmental stages of the parasite was assessed. TcNaa10 was mainly seen around the nucleus in midlog and stationary epimastigotes (Figure 3(b)). In trypomastigotes, TcNaa10 appeared exclusively around the nucleus (Figure 3(b)). The staining profile of TcNaa10 in amastigotes, meanwhile, was restricted to the periphery of the cell (Figure 3(b)). In all the life cycle stages, TcNaa15 appeared to predominantly localize to the cell periphery (Supplementary Figure S3B). The cytoplasmic labeling disappeared in the metacyclic stages as TcNaa15 localized to the kinetoplast (Supplementary Figure S3B). Tissue culture trypomastigotes exhibited a more diffuse cytoplasmic localization and expression was reduced to a structure resembling the remaining short flagellum in amastigotes (Supplementary Figure S3B).

### 3.6. TcNaa30/TcNaa38 and TcNaa10/TcNaa15 Co-Sediment with the Ribosome

**Figure 4:**
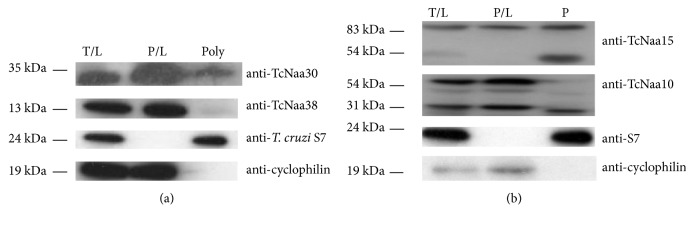
Association of TcNaa30/TcNaa38 and TcNaa10/TcNaa15 with the ribosome. (a) Membrane was incubated with anti-TcNaa30 and anti-TcNaa38. Total cell lysate (T/L), supernatant postultracentrifugation (P/L) and polysomes (Poly) were loaded. As controls, anti-*T. cruzi* S7 (specific for the ribosome) and anti-*T. cruzi* cyclophilin A (nonribosomal) were used. Molecular size markers in kDa are indicated on the left. (b) Membrane was incubated with anti-TcNaa10 and TcNaa15. Loading control as mentioned in Figure 4(a).

We examined the TcNaC cosedimentation with the ribosome through a sucrose cushion, and, as shown in (Figure 4(a)), TcNaa30 is present in both the ribosomal and nonribosomal fractions. A smaller amount of TcNaa38 could also be observed in the polysome fraction. The results for TcNatA (Figure 4(b)) showed the presence of TcNaa10 and TcNaa15 in both the ribosomal and nonribosomal fractions. Anti-TcNaa15 detected an additional band of approximately 54 kDa in the ribosomal fraction. In contrast, anti-TcNaa10 detected a band of about the same size in the nonpolysome fraction. The identity of the extra band is not known. Taken together, the ribosomal cosedimentation results indicated that the TcNatA and TcNatC proteins might associate with the ribosomes.

### 3.7. T. cruzi NatC and TcNatA Subunits Interact In Vivo and In Vitro

**Figure 5:**
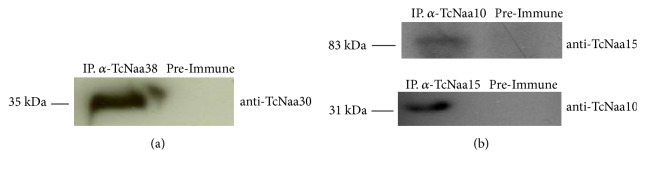
TcNatC and TcNatA protein interaction. (a) Immunoprecipitation (IP) of TcNaa30. The parasite lysate was incubated with anti-TcNaa38. As a control, the lysate was incubated with rabbit sera (preimmune). The blot was analyzed with anti-TcNaa30. Molecular weight marker in kDa is indicated. (b) Coimmunoprecipitation assays of TcNaa10 with TcNaa15 protein. IP with preimmune sera was used as a control. Western blots of the immunoprecipitated samples were probed with rabbit anti-TcNaa10 and anti-TcNaa15.

Human orthologs of TcNaa30, TcNaa35, and TcNaa38 form a stable complex* in vivo* [8]. To investigate whether TcNaa30 and TcNaa38 formed a stable complex in* T. cruzi*, immunoprecipitation using anti-TcNaa30 and anti-TcNaa38 was performed. Immunoprecipitation with anti-TcNaa30 was unsuccessful, but using anti-TcNaa38 we were able to immunoprecipitate TcNaa30 (Figure 5(a)). Though further study is required, this indicates that these proteins physically interact in* T. cruzi*, either directly or through another protein, for example, the ribosome complex. Likewise, immunoprecipitation showed that anti-TcNaa10 was able to immunoprecipitate TcNaa15 (Figure 5(b)) upper panel. By reciprocal immunoprecipitation, anti-TcNaa15 was able to pull down TcNaa10 (Figure 5(b)), lower panel. This analysis suggests that the TcNaa10 and TcNaa15 interact* in vivo *in the same way as yeast and human orthologs of the TcNaa10 and TcNaa15 form a stable complex* in vitro* and* in vivo* [7, 9, 27].

### 3.8. In Vitro N*α*-Acetyltransferase Assay

**Figure 6:**
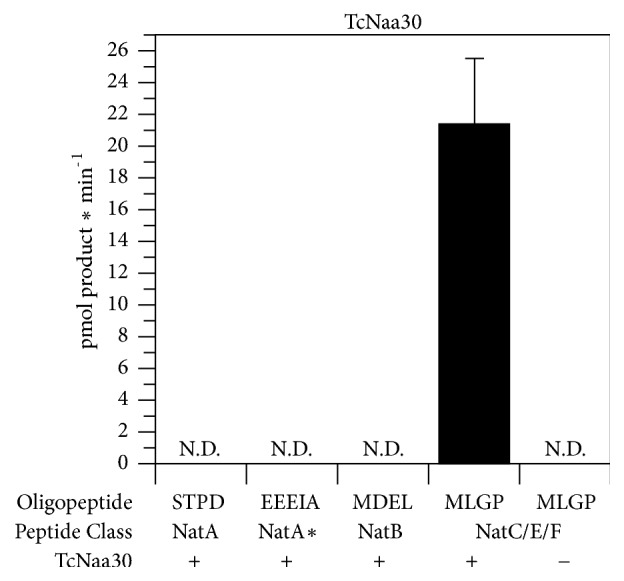
*In vitro* acetyltransferase activity of TcNaa30. GST-TcNaa30 was incubated with acetyl-CoA (300 mM) and selected oligopeptides (300 mM) for 30 min at 37°C. dH2O was used as negative control. The amount of acetylated peptide was determined with reverse phase HPLC. Oligopeptide names indicate the first four amino acids from the N-terminus. ND represents nondetectable. *∗* indicates that NatA can also posttranslationally acetylate acidic N termini, for example, *γ* actin.

In order to investigate the substrate specificity of TcNatC and TcNatA, we performed an* in vitro* Nt-acetylation assay where purified recombinant protein (GST-TcNaa30) was incubated with synthetic peptides representing substrates for different classes of NATs (NatA-NatE). As shown in (Figure 6), TcNaa30 preferentially acetylates a peptide with a hydrophobic N-terminal sequence of MLGP, which corresponds to a typical NatC/E/F substrate in humans. We also attempted to assess TcNaa10 enzymatic activity and whether the TcNaa10 substrate preferences are identical to those in human cells in a similar way as above. Though there was an indication of Naa10 activity, preferentially acetylating the synthetic peptide sequences STPD and EEEIA (not shown), representing human NatA substrates, no reproducible activity was found.

### 3.9. Effect of Knock down of Predicted T. brucei NatC Catalytic Subunit by RNAi

RNAi was carried out on the* T. brucei* equivalent of the TcNaa30 gene. RNAi was induced in* Trypanosoma brucei brucei* 427 using tetracycline. For the wild type, tetracycline had no effect on their viability (Supplementary Figure S5A).

**Figure 7:**
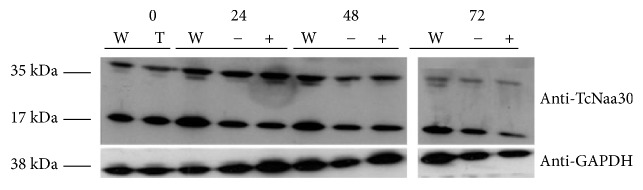
Phenotype of the knock-down of putative* T. brucei* Naa30 by RNAi. TcNaa30 protein expression analysis of wild type (W), transformant (T), noninduced (-), and induced (+) cells by western blotting. Cells were counted, washed, and dissolved in sample buffer and immediately boiled. About equal amount of each sample was used in the experiment. Note that a band of about 17 kDa, whose identity is not known, was also identified. GAPDH was used as loading control.

We observed a reduction in parasite growth in both the induced and noninduced after 48 h and 72 h (Supplementary Figures S5B and S5C). This most likely indicated that the RNAi vector was leaky, or the more unlikely possibility that the vector itself causes the effect. An RT-PCR assay (Supplementary Figure S5D) indicated that at 48 h after induction, there was a decrease in the levels of endogenous mRNA in the induced and noninduced transformant cell compared to the wild type. Western blotting using anti-*T. cruzi* NatC (TcNaa30) showed that, especially after 48 h, there was lower protein expression in the noninduced and induced cells compared to the wild type (Figure 7).

## 4. Discussion

We here describe the molecular cloning and characterization of the predicted* T. cruzi* NatC and* T. cruzi* NatA N*α*-acetyltransferase protein complexes. We found that protein Nt-acetylation by* T. cruzi* NatC and NatA was similar to what has been described in other eukaryotes. It appears that the expression profile of TcNatA and TcNatC in different parasite life cycle is not uniform. But, how this translates to the distinct parasite morphologies and biology is not clear. Similar to expression, the localization profile of TcNatC and TcNatA proteins by immunofluorescence in the different life cycle forms are diverged. The functional significance of these is speculated. Similar staining patterns were observed [16, 28, 29] for human Naa40/NatD and other NATs proteins. Given the divergent expression and localization of the TcNatC and TcNatA proteins, it is tempting to speculate that, the given protein is located at a particular compartment at a given time to carry biological tasks. Considering localization of* T. brucei* (Tb927.7.2360), a similar gene to TcNaa30 by GFP-tagged version [30], the* T. brucei* gene N-terminally and C-terminally tagged versions are distributed throughout the cell. Localization of TcNaa30 in our hands is predominantly distributed in the cytoplasm, suggesting differential biological function in trypanosomes. Further analyses are needed to confirm this hypothesis.

TcNatC and TcNatA proteins physically interact with each other and it is plausible that this interaction takes place in the cytoplasm as suggested by their possible ribosomal cosedimentation. Possibly, the proteins in some cases carry out their function independently of each other as suggested in other organisms [16] and that they may have specific functions depending on the parasite life cycle stage. TcNatC/TcNatA proteins may also have other functions independent of the NAT-activity as suggested in other species [15].

The biological significance of posttranslational modification of proteins, especially acetylation, in trypanosomes is relatively unexplored [31]. We predicted the TcNatC substrates profile and detected many parasite-specific proteins that lack homologues in humans (Table 2). For TcNatA substrates, the predictions include hypothetical proteins, as well as mucin-associated surface protein (MASP) and mucin proteins (not shown). The MASP gene family is preferentially expressed in the trypomastigote [32]. Moreover, it is exposed to the host immune system and possibly used by the parasite during infection [32]. Another noticeable predicted* T. cruzi* NatC and TcNatA substrate is transsialidase (TS), a polymorphic surface enzyme used by the parasite during infection [33]. Taken together, it can be speculated that Nt-acetylation, if lost, could simultaneously affect many surface antigens including TS, or many parasite-specific functions and cellular processes that are important for pathology.

For the extracellular parasite* T. brucei*, some proteins used by the parasite to evade the host immune system were predicted as possible substrates for TbNatC (not shown). These include receptor-like adenylate cyclases [34], variant surface glycoprotein, and an expression site- associated gene [35]. Study of the N-terminal acetylome by proteomic methods in trypanosomes [31] confirms our prediction that Nt-acetylation state in these organisms is common. Further studies are required for a complete understanding of which cellular machineries are regulated this way and how this is important for the life of the parasite.

In yeast, human, and plants, the biological significance of NatC knockdown has been investigated [8, 36, 37]. These studies point towards loss of cell viability if NatC is depleted. The NatA protein complex was found to be essential for cell survival in* T. brucei* [18]. Given the sequence identity and the similar predicted ligand binding and active sites, it is likely that NatA is essential in all trypanosomatids. In this study, silencing of the* T. brucei* NatC predicted catalytic subunit by RNAi suggests that the protein may be important to the parasite, though there was minor reduction of the predicted protein band in the blot in the transfected cells compared with the control. Another system for conditional knockouts for trypanosomatids such as CRISPR/Cas9 could be tested to ascertain our observation in this study or perform genome-scale RNAi [38] by silencing the parasites NATs catalytic subunits and phenotypes assessed. It is clear though that these are basic, important functions that are of interest for gene function and regulation as well as for possible drug target testing.

Collectively, identification of all the NATs in* T. cruzi, *analyzing substrates preferences, and proteomic study of Nt-acetylation in all the developmental stages will narrow the gaps in knowledge of the parasite biology.

## Figures and Tables

**Table 1:** TcNatC and TcNatA genes. *∗* denotes genes investigated in this study.

TcNat	Gene	CL Brener haplotype
TcNatC catalytic subunit (TcNaa30)	Tc00.1047053511809.120 (Tc9.120)	Non- Esmeraldo-like *∗*
	Tc00.1047053511811.30 (Tc1.30)	Esmeraldo- like
TcNatC auxiliary subunit (TcNaa35)	Tc00.1047053511311.80 (Tc1.80)	Esmeraldo- like
	Tc00.1047053511755.119 (Tc5.119)	Non- Esmeraldo-like *∗*

TcNatC auxiliary subunit (TcNaa38)	Tc00.1047053507209.10 (Tc9.10)	Non- Esmeraldo-like *∗*
TCNatA catalytic subunit (TcNaa10)	TcCLB.506227.230 (Tc7.230)	Esmeraldo-like *∗*
TCNatA auxiliary subunit (TcNaa15)	TcCLB.504163.110 (Tc3.110)	Esmeraldo-like
	TcCLB.510301.80 (Tc01.80)	Non-Esmeraldo-like *∗*

**Table 2:** Some estimated number of predicted TcNaa30 substrates for CL Brener haplotypes based on Met-Leu, Met-Ile, Met-Phe, and Met-Tyr N-termini.

	Non-Esmeraldo	Esmeraldo
Total genes	1461	1328
hypothetical	830	774
transsialidase	248	203
mucin TcMUCII	2	2
MASP	4	3

## Data Availability

The data used to support the findings of this study are included within the article and within the supplementary information file(s).

## Supplementary Materials

Supplementary Materials
** Supplementary Figure** **1:** alignment of TcNaa35/TcNaa38 and TcNaa10/TcNaa15 with orthologous sequences from other eukaryotes.** A:** comparison of predicted TcNaa35 with other proteins. The tick line indicates predicted Mak10 domain region. The most conserved aa are represented by a star (*∗*). Note that only part of the alignment is displayed. Polypeptides used are* Leishmania major* (XP_001682355.1),* T. cruzi* (XP_814406.1),* T. brucei* (XP_844906.1), Plant (NP_001118295.1), human (SP ∣Q5VZE5), and Yeast (NP_579858.1).** B:** TcNaa38 sequence alignment with selected proteins. *∗* denotes the most conserved aa. Sequences included in the alignment are yeast (NP_010946.3), human (NP_001317040.1),* Leishmania major *(XP_003722006.1),* T. cruzi *(XP-808107.1), and* T. brucei* (XP_951644.1).** C:** comparison of TcNaa10 sequence with selected eukaryotes. The overlined indicates the consensus sequence RXXGXA for NatA, where acetyl–CoA binds. The two arrows indicate the active sites and a symbol* ♯* is the predicated possible lysine autoacetylation site. Used sequences in the alignment are yeast (NP_011877.1), human (NP_003482.1),* Leishmania major *(XP_001681770.1),* T. cruzi* (XP_817467.1), and* T. brucei* (XP_828529.1).** D: **TcNaa15 sequence comparison with putative homologues from other species. Polypeptides used in the alignment are* Leishmania major* (XP_001686706.1),* T. cruzi* (XP_807954.1),* T. brucei* (XP_822811.1), human (NP_476516.1), and yeast (END60314.1). *∗* indicates residues, which are highly conserved.** Supplementary Figure ****2:** recombinant protein production and analysis on 10 % SDS-PAGE.** A:** recombinant GST-TcNaa35.** B:** GST-TcNaa38.** C:** GST-TcNaa10 and** D:** GST-TcNaa15. Samples loaded in the different lanes are as follows: Lane 1, molecular mass in kDa. Lane 2, noninduced (NI). Lane 3, induced (IND). Lane 4, pellet (P). Lane 5, supernatant (Sup). Lane 6, semipurified (S/Purified).** Supplementary Figure ****3:** localization of TcNaa38 and TcNaa15. Numbers 1 to 5 denote midlog epimastigotes, stationary epimastigotes, metacyclic trypomastigotes, trypomastigotes, and amastigotes, respectively.* T. cruzi* four developmental stages were immunolabelled with** A,** anti-TcNaa38 and** B,** anti-TcNaa15. The nucleus and kinetoplast were visualized using DAPI stain (N+K), scale bars = 5 *μ*m.** Supplementary Figure ****4:** localization of TcNaa30 and TcNaa38 in internal amastigotes. Parasites were allowed to invade Vero cells and divide. Cells were immunolabelled with anti-TcNaa30 and anti-TcNaa38 (green) and counter labeled with DAPI (blue) to visualize the nucleus and kinetoplast, scale bars = 10 *μ*M.** Supplementary Figure ****5:** phenotype of the knock down of putative* T. brucei* Naa30 by RNAi showing growth curves and mRNA level;** A, **wild type;** B,** transfected;** C,** the parasite growth of the transfectants compared to that of the wild type (blue). Cells at a density of 2.5x10^4^/ml were grown in the absence (-**Tet**) or presence (**+Tet**) of tetracycline (100ng/ml) over a period of 72 h. The result is a representative of the mean parasite growth of* T. b. brucei* 427 obtained from three independent experiments.** D,** gene expression analysis of wild type (W), noninduced (-), and induced (**+**) cells using Reverse Transcriptase PCR. The products were separated on a 2 % agarose gel alongside a 1kb DNA ladder and visualized using ethidium bromide staining. Shown is days 0-48 h and day 72 h post-RNAi induction. Actin (700bp) was amplified as an internal control alongside NatC catalytic subunit (573bp). The cell densities used for days 0, 24, 48, and 72 h from which RNA was isolated were 1x10^5^/ml, 2.73x10^5^/ml, 3.75x10^5^/ml, and 4.7x10^5^/ml, respectively.
