# Supplementary material for: Characterization of Evolutionarily Conserved Trypanosoma cruzi NatC and NatA-N-Terminal Acetyltransferase Complexes
Source: J Parasitol Res. 2019 Mar 6;2019:6594212. doi: 10.1155/2019/6594212 (PMC6431383; doi:10.1155/2019/6594212)
Supplement: Supplementary Materials — Supplementary Figure 1: alignment of TcNaa35/TcNaa38 and TcNaa10/TcNaa15 with orthologous sequences from other eukaryotes. A: comparison of predicted TcNaa35 with other proteins. The tick line indicates predicted Mak10 domain region. The most conserved aa are represented by a star (∗). Note that only part of the alignment is displayed. Polypeptides used are Leishmania major (XP_001682355.1), T. cruzi (XP_814406.1), T. brucei (XP_844906.1), Plant (NP_001118295.1), human (SP ∣Q5VZE5), and Yeast (NP_579858.1). B: TcNaa38 sequence alignment with selected proteins. ∗ denotes the most conserved aa. Sequences included in the alignment are yeast (NP_010946.3), human (NP_001317040.1), Leishmania major (XP_003722006.1), T. cruzi (XP-808107.1), and T. brucei (XP_951644.1). C: comparison of TcNaa10 sequence with selected eukaryotes. The overlined indicates the consensus sequence RXXGXA for NatA, where acetyl–CoA binds. The two arrows indicate the active sites and a symbol ♯ is the predicated possible lysine autoacetylation site. Used sequences in the alignment are yeast (NP_011877.1), human (NP_003482.1), Leishmania major (XP_001681770.1), T. cruzi (XP_817467.1), and T. brucei (XP_828529.1). D: TcNaa15 sequence comparison with putative homologues from other species. Polypeptides used in the alignment are Leishmania major (XP_001686706.1), T. cruzi (XP_807954.1), T. brucei (XP_822811.1), human (NP_476516.1), and yeast (END60314.1). ∗ indicates residues, which are highly conserved. Supplementary Figure 2: recombinant protein production and analysis on 10 % SDS-PAGE. A: recombinant GST-TcNaa35. B: GST-TcNaa38. C: GST-TcNaa10 and D: GST-TcNaa15. Samples loaded in the different lanes are as follows: Lane 1, molecular mass in kDa. Lane 2, noninduced (NI). Lane 3, induced (IND). Lane 4, pellet (P). Lane 5, supernatant (Sup). Lane 6, semipurified (S/Purified). Supplementary Figure 3: localization of TcNaa38 and TcNaa15. Numbers 1 to 5 denote midlog epimastigotes, stationary epimastigot [file 6594212.f1.pdf]

A

```
XP_001682355.1            ----------------------------CDLNDTWEDVLTAFNDCLK-GESTWSCHCLDG   31
XP_814406.1               --------------------------MACDLDETWVECMGEFKEFIGKTGNPWRCYTCEG   34
XP_844906.1               --------------------------MLCALDDTWADCLDEFKLALSSTGNTWKCESLAG   34
NP_001118295.1            --MQSVREDEDSSSPIHHDSKTSSSIPSGDNNSVWADVSPLLSAACSDLQEGE-LINGDN   57
sp|Q5VZE5|NAA35_HUMAN     MVMKASVDDDDSGWEL------SMPEKMEKSNTNWVDITQDFEEACRELKLGE-LLHDKL   53
NP_579858.1               MVMKAAVDDDASGWEL------NVPEKMEKSSTSWVDITQDFEDACRELKLGE-LLHDKL   53
                                                         .  * :    :.

XP_001682355.1            IDKEVILSAPEIMDPKTDSGFNCEEI----YSLSHLLKTEEVPS----AATVSTEAALLD   83
XP_814406.1               VEKETMLSAPEVMDAKTDPGCGYGSI----CSLNCLLKDGTIPS----AATLTG-EALLD   85
XP_844906.1               VDKEAMLSAPEVMDAKTDPGCGYAQI----RSLNDLLAKGDIPS----AATLQG-QELLD   85
NP_001118295.1            FNLFAAMSALEIMDPKMDSGMVSTFYS-----IDEAIESGFAPVPISSDSTVN-VQSIID   111
sp|Q5VZE5|NAA35_HUMAN     FGLFEAMSAIEMMDPKMDAGMIGNQVNRKVLNFEQAIKDGTIK-----IKDLT-LPELIG   107
NP_579858.1               FGLFEAMSAIEMMDPKMDAGMIGNQVNRKVLNFEQAVKDGTIK-----IKDLS-LPELIG   107
                          .     :** *:** * * *            :.  :              :     ::.

XP_001682355.1            VMDYIHVKELTYLQGYSLTQSYLDFPYFLRMDLLKGQNSTLHAYCRGVLRSLDCVLHAVF   143
XP_814406.1               VMDLIHLKELNYLQGFSLTSGCLEFSYFFCMDLLKEQNLTLYTYCRALARCIDLTTRAVM   145
XP_844906.1               VMDLIILAKELQYLQGFSLTSGCLAFPYFFKMDLLKEQNPVLHAYCRGVVRTVEIVLRAVM   145
NP_001118295.1            IMDHLLACEATWHMGHSLAQTVFSCIYVLRPERTSSQ-ALLHSYCRVIRATCRAVVSVVS   170
sp|Q5VZE5|NAA35_HUMAN     IMDTCFCCLITWLEGHSLAQTVFTCLYIHNPDFIEDP-AM-KAFALGILKICDIAREKVN   165
NP_579858.1               IMDTCFCCLITWLEGHSLAQTVFTCLYIHNPDFIEDP-AM-KAFALGILKICDIAREKVN   165
                          :**        :  *.**:.  :   *.   :  .       ::.  :      .   *
```

B

```
NP_010946.3          ---------IDYKLRVLTQDGRVYIGQLMAFDKHMNLVLNECIEERVPKTQLDKLRPRKD     51
NP_001317040.1       -ARQQLEALLNKTMRIRMTDGRTLVGCFLCTDRDCNVILGSAQEFLKPSDSF--------     51
XP_003722006.1       MGKYTMLHNINKVLCVMLDDGRTVTGKLLVFDKHMNVVLGDAVEERPQSKKMAEE-----     55
XP_808107.1          MGRESMLHNINRTLRIVLIDGRELTGKLLVYDRHMNVVLGDATESREETKKMKEA-----     55
XP_951644.1          MGHQNMLHNINRTLRVTLVDGREMTGKMLLFDKFMNVVLADTVETRKETKKMKDA-----     55
                              ::  : :   ***   * ::  *:  *::* .  *    . .:

NP_010946.3          SKDGTTLNIKVEKRVLGLTILRGEQILSTVV-----------------------------     82
NP_001317040.1       --------SAGEPRVLGLAMVPGHHIVSIEV-----------------------------     74
XP_003722006.1       --------GVSSKRQLGLILLRGEHVVSVTVMKDSENGGSGAVAN------FGGAPKLAK     101
XP_808107.1          --------GISPQRSLGLVLLRGVHVISVNVLGASENNGDGEGKTKGQPANFEKAPRAKV     107
XP_951644.1          --------GISPQRKLGMILLRGEYVVAVSVLKDN------VSEDKAQPANFESATREKL     101
                                  * **: :: *  :::  *
```

C

```
                                                                      #
NP_011877.1          TYRRMGIAENLMRQALFALREVHQAEYVSLHVRQSNRAALHLYRDTLAFEVLSIEKSYYQ     180
NP_003482.1          SHRRLGLAQKLMDQASRAMIENFNAKYVSLHVRKSNRAALHLYSNTLNFQISEVEPKYYA     139
XP_001681770.1       SHRKLGIASRVMRATMKEMDAEYGAHYCSLHVRKTNDAALHLYQDTLGFRCVGVEEKYYM     140
XP_817467.1          THRRLGIASRVMRASMKEMEKEYDANYCSLHVRKTNDAALHLYQETLGFRCANVEKGYYV     140
XP_828529.1          THRRLGIASRVMNAALHEMEHEYDANFCSLHVRKTNDAALHLYQNTLNFRCANVESKYYV     140
                     ::*::*:*..:*  :    :    . *.: *****::* ****** :** *.   :*  **

NP_011877.1          DGEDAYAMKKVLK-----------------------------------------------     193
NP_003482.1          DGEDAYAMKR--------------------------------------------------     149
XP_001681770.1       DEEDAYHMKSFFHQANPGSYVDDHKRLIRK------------------------------     170
XP_817467.1          DEEDAFHMKKFFKGPNPGLYVAANRQLVRQQNSAAAAVTGMAALQRNGGTAPPPPPPSSS     200
XP_828529.1          DEEDAYHMKRFFKGTNPGFYVTESRQLVRQPNT---------------------------     173
                     * ***: **
```


```
NP_011877.1          TYRRMGIAENLMRQALFALREVHQAEYVSLHVRQSNRAALHLYRDTLAFEVLSIEKSYYQ     180
NP_003482.1          SHRRLGLAQKLMDQASRAMIENFNAKYVSLHVRKSNRAALHLYSNTLNFQISEVEPKYYA     139
XP_001681770.1       SHRKLGIASRVMRATMKEMDAEYGAHYCSLHVRKTNDAALHLYQDTLGFRCVGVEEKYYM     140
XP_817467.1          THRRLGIASRVMRASMKEMEKEYDANYCSLHVRKTNDAALHLYQETLGFRCANVEKGYYV     140
XP_828529.1          THRRLGIASRVMNAALHEMEHEYDANFCSLHVRKTNDAALHLYQNTLNFRCANVESKYYV     140
                     ::*::*:*..:*  :    :    . *.: *****::* ****** :** *.   :*  **

NP_011877.1          DGEDAYAMKKVLK-----------------------------------------------     193
NP_003482.1          DGEDAYAMKR--------------------------------------------------     149
XP_001681770.1       DEEDAYHMKSFFHQANPGSYVDDHKRLIRK------------------------------     170
XP_817467.1          DEEDAFHMKKFFKGPNPGLYVAANRQLVRQQNSAAAAVTGMAALQRNGGTAPPPPPPSSS     200
XP_828529.1          DEEDAYHMKRFFKGTNPGFYVTESRQLVRQPNT---------------------------     173
                     * ***: **
```


D

```
XP_001686706.1       YSNREYSKALRTSECILRVV--PDHVDTFAGRGLVLYNMERQEEGYESIKQAI---LLNP     55
XP_807954.1          FDAREYAKALRCADAILAVI--PSHADTIAMRGLTLHHLDRREEGHLAIKEAI---ELNI     55
XP_822811.1          FDAREYAKGLRTADSILSVV--PNHADTLALKGLTLHHMGRKEEGREIIESAL---GFND     55
NP_476516.1          YEHKQYRNGLKFCKQILSNPKFAEHGETLAMKGLTLNCLGKKEEAYELVRRGL---RNDL     57
EDN60314.1           YEGKQYKKSLKLLDAILKKD--GSHVDSLALKGLDLYSVGEKDDAASYVANAIRKIEGAS     58
                     :.  ::* :.*:   . **       .* :::* :** *   :  .:::.    :   .:

XP_001686706.1       KSMVAWHALGMCQRLDKKFGEAVKAFKRALTFDPANTEVLRDLASACIQVRDWPLFLEAR     115
XP_807954.1          NSTMAWHSLGMCHRAEKNYPEAIKAFKKAHQTDPTNANVLRDLSSVCVQVRDWEQFVETR     115
XP_822811.1          TSTVVWHSLGMCHRADDNHVEALHAFQKAHEYGPSNVNVLRDISSICVQLREWEQFVDVR     115
NP_476516.1          KSHVCWHVYGLLQRSDKKYDEAIKCYRNALKWDKDNLQILRDLSLLQIQMRDLEGYRETR     117
EDN60314.1           ASPICCHVLGIYMRNTKEYKESIKWFTAALNNGSTNKQIYRDLATLQSQIGDFKNALVSR     118
                      * :  *  *:  *   .:. *::: :  *    .  * :: **::    *: :      *

XP_001686706.1       EKMVTAKASVRANWVALSCGHRILGHSRIAAAVMDTMTSIMDA---GDNPVEVSEAQLYR     172
XP_807954.1          QKMVTLKAGVRANWIALSCGHRMMGHTELAAAVIDVMTSIMDA---GENVVEKSEVFLYL     172
XP_822811.1          RKMVTLRPGVRANWIALSCGHRMLGNKELAAAVIDVMTTIMEA---GDNRAEKSEVRLYQ     172
NP_476516.1          YQLLQLRPAQRASWIGYAIAYHLLEDYEMAAKILEEFRKTQ-QTSPDKVDYEYSELLLYQ     176
EDN60314.1           KKYWEAFLGYRANWTSLAVAQDVNGERQQAINTLSQFEKLAEGKISDSEKYEHSECLMYK     178
                      :      . **.* . : .   :   . . *    :. : .          ..   * **  :*
```

Supplementary Figure 1: Alignment of TcNaa35/TcNaa38 and TcNaa10/TcNaa15 with orthologous sequences from other eukaryotes. **A;** Comparison of predicted TcNaa35 with other proteins. The tick line indicates predicted Mak10 domain region. The most conserved aa are represented by a star (*). Note that only part of the alignment is displayed. Polypeptides used are: *Leishmania major* (XP_001682355.1), *T. cruzi* (XP_814406.1), *T. brucei* (XP_844906.1), Plant (NP_001118295.1), human (SP|Q5VZE5) and Yeast (NP_579858.1). **B;** TcNaa38 sequence alignment with selected proteins. * Denote the most conserved aa. Sequences included in the alignment are: Yeast (NP_010946.3), Human (NP_001317040.1), *Leishmania major* (XP_003722006.1), *T. cruzi* (XP-808107.1) and *T. brucei* (XP_951644.1)

C; Comparison of TcNaa10 sequence with selected eukaryotes. The over lined indicates the consensus sequence RXXGXA for NatA, where acetyl –CoA binds. The two arrows indicate the active sites and a symbol ♯ is the predicated possible lysine auto acetylation site. Used sequences in the alignment are: Yeast (NP_011877.1), Human (NP_003482.1), *Leishmania major* (XP_001681770.1), *T. cruzi* (XP_817467.1) and *T. brucei* (XP_828529.1). **D;** TcNaa15 sequence comparison with putative homologues from other species. Polypeptides used in the alignment are: *Leishmania major* (XP_001686706.1), *T. cruzi* (XP_807954.1), *T. brucei* (XP_822811.1), Human (NP_476516.1 and Yeast (END60314.1. * Indicates residues, which are highly conserved.

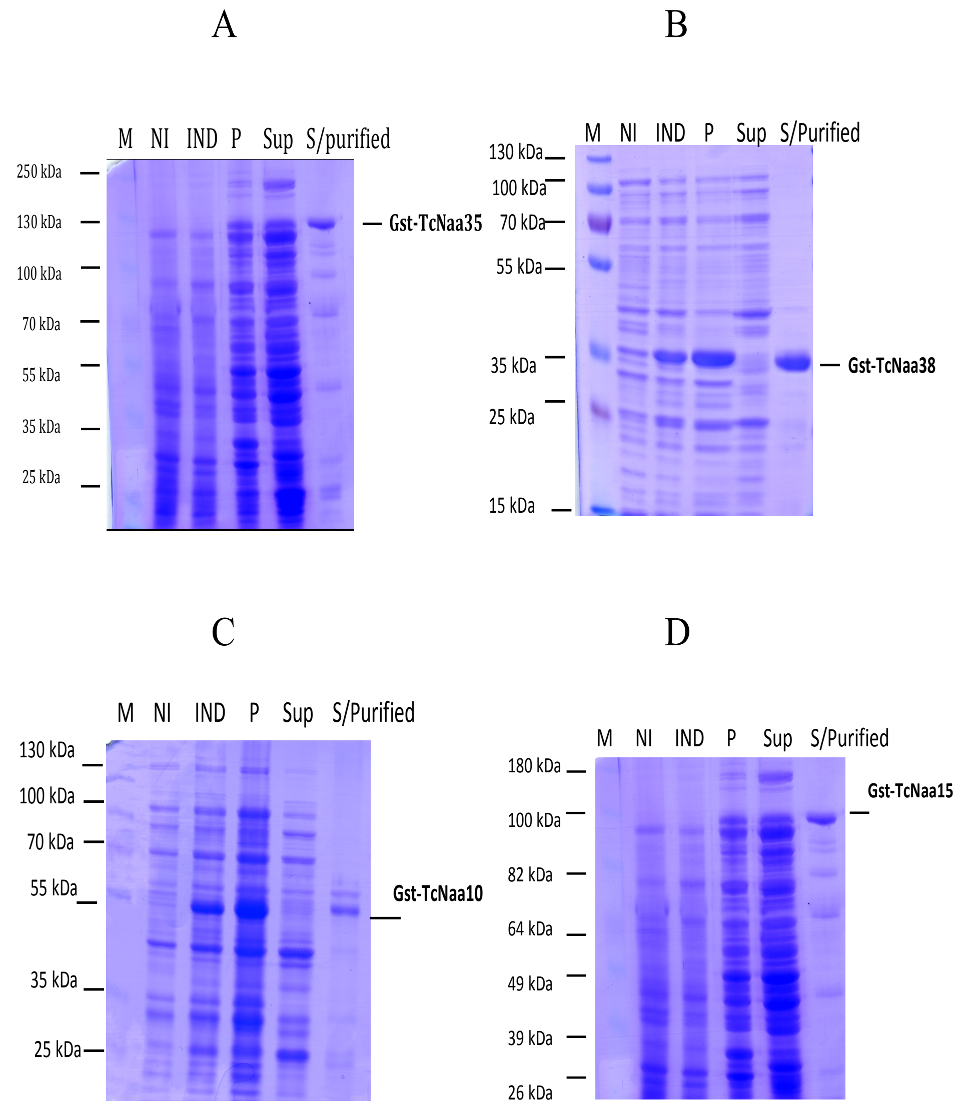

Supplementary Figure 2: Recombinant protein production and analysis on 10 % SDS-PAGE. **A;** Recombinant GST-TcNaa35. **B;** GST-TcNaa38. **C;** GST-TcNaa10 and **D;** GST-TcNaa15. Samples loaded in the different lanes are as follows, Lane1; Molecular mass in kDa. Lane 2; Non-induced (NI). Lane 3; Induced (IND), Lane 4; Pellet (P). Lane 5; Supernatant (Sup). Lane 6; Semi Purified (S/Purified).

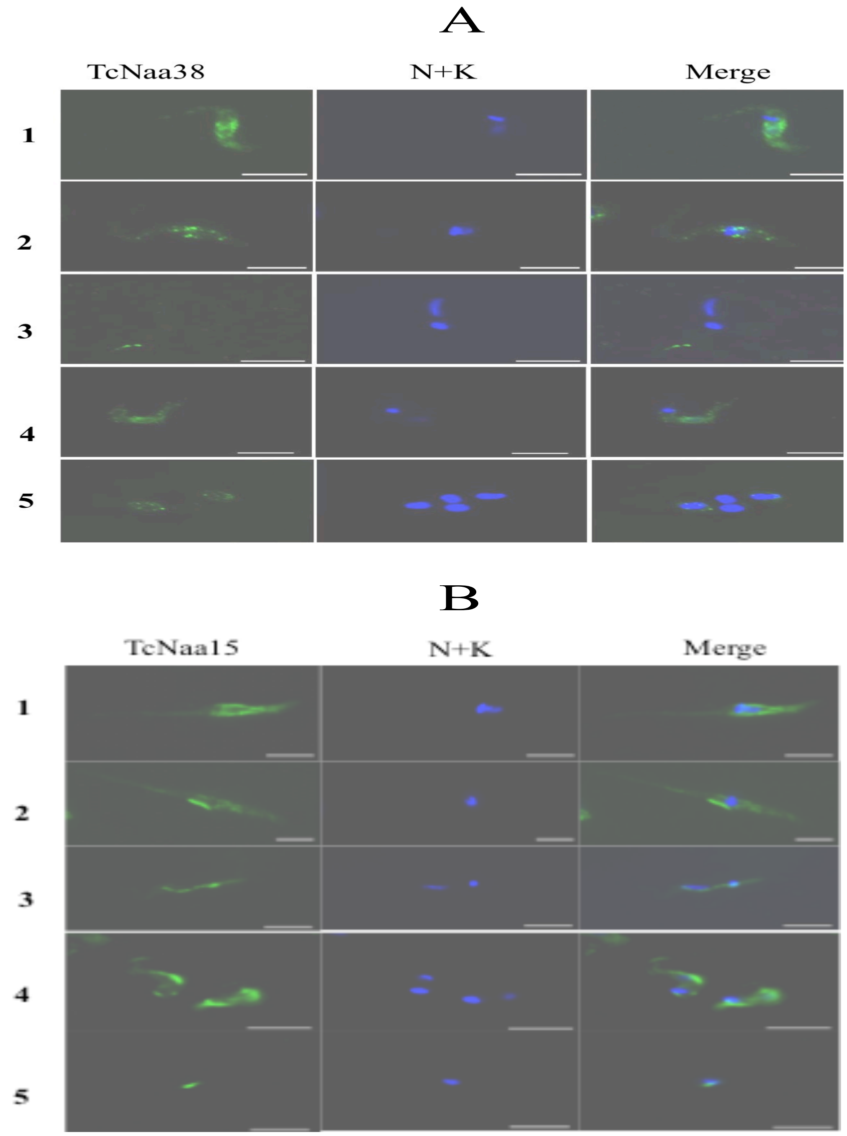

Supplementary Figure 3: Localization of TcNaa38 and TcNaa15. Number 1 to 5 denotes, Midlog epimastigotes, Stationary epimastogotes, Metacyclic trypomastigotes, Trypomastigotes, and Amastigotes, respectively. *T. cruzi* four developmental stages were immunolabelled with, **A;** anti-TcNaa38 and **B;** anti-TcNaa15. The nucleus and kinetoplast were visualized using DAPI stain (N+K), scale bars = 5 µm.

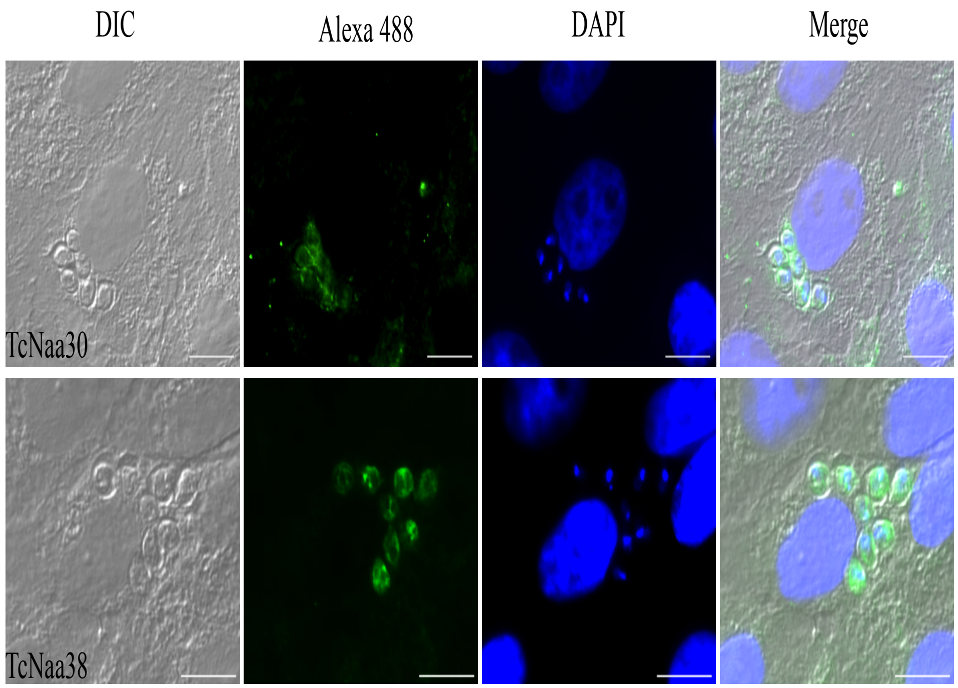

Supplementary Figure 4: Localization of TcNaa30 and TcNaa38 in internal amastigotes. Parasites were allowed to invade Vero cells and divide. Cells were immunolabelled with anti - TcNaa30 and anti- TcNaa38 (green) and counter labeled with DAPI (blue) to visualize the nucleus and kinetoplast, scale bars = 10 μM.

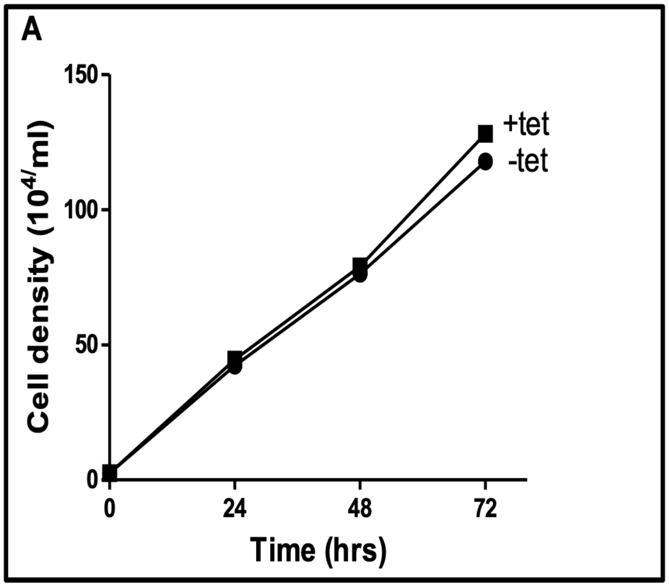
A
150
100
50
0
Cell density (10^4/ml)
0
24
48
72
Time (hrs)
+tet
-tet

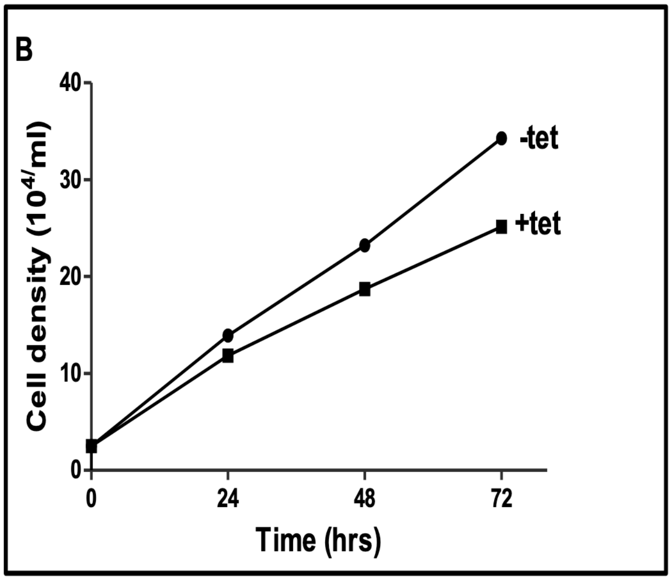
B
40
30
20
10
0
Cell density (10^4/ml)
0
24
48
72
Time (hrs)
-tet
+tet

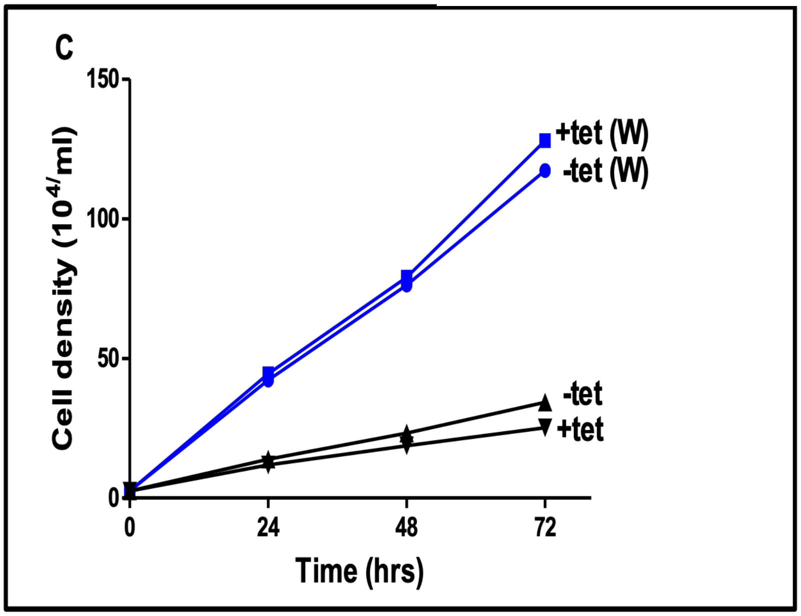
C
150
100
50
0
Cell density (10^4/ml)
0
24
48
72
Time (hrs)
+tet (W)
-tet (W)
-tet
+tet

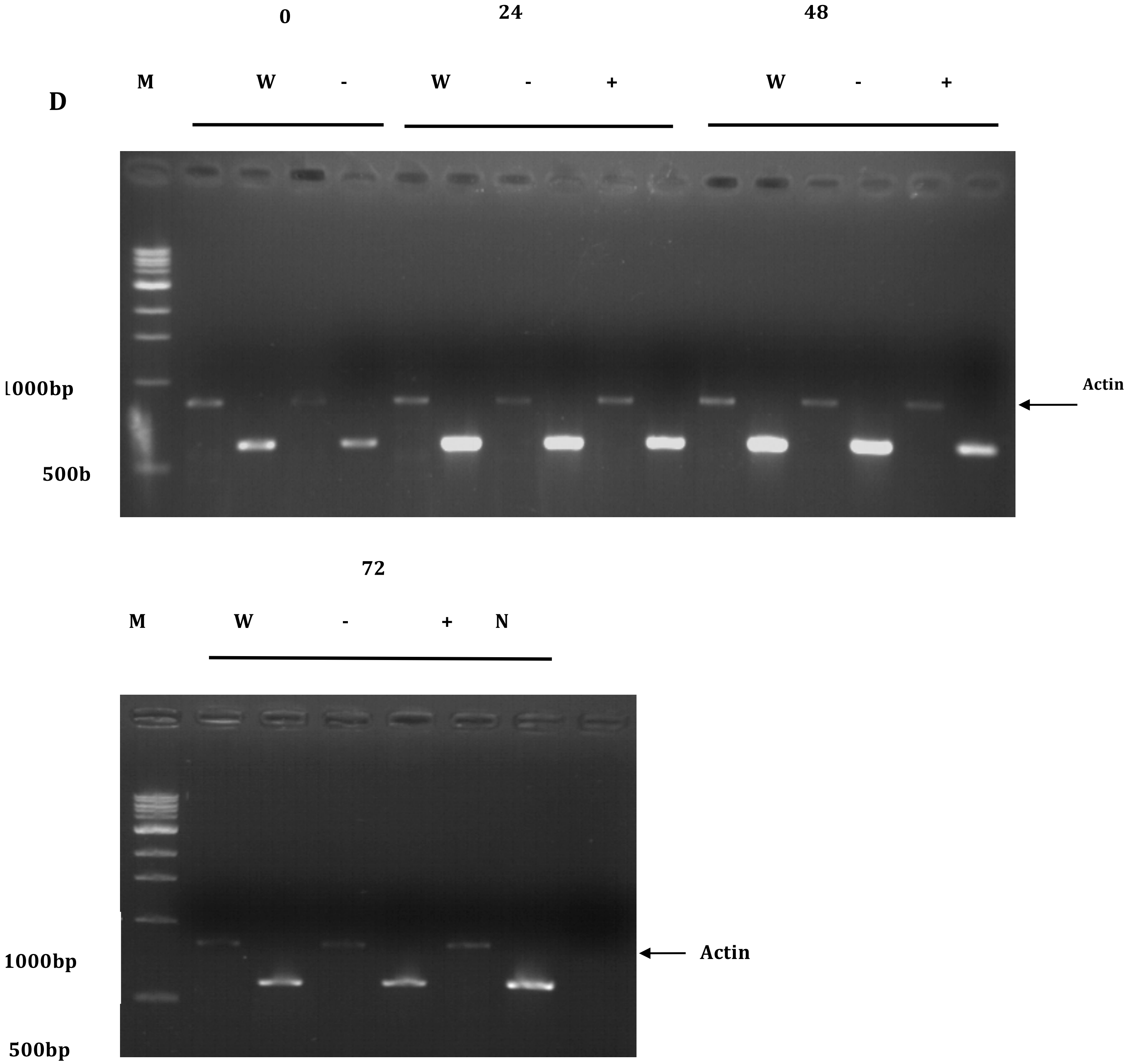
D
0
24
48
M
W
-
W
-
+
W
-
+
1000bp
500b
Actin
72
M
W
-
+
N
1000bp
500bp
Actin

Supplementary Figure 5: Phenotype of the knock down of putative *T. brucei* Naa30 by RNAi showing growth curves and mRNA level, **A;** Wild type, **B;** Transfected, **C;** The parasite growth of the transfectants compared to that of the wild type (blue). Cells at a density of 2.5×10^4/ml were grown in the absence (-**Tet**) or presence (+**Tet**) of tetracycline (100ng/ml) over a period of 72h. The result is a representative of the mean parasite growth of *T. b. brucei* 427 obtained from three independent experiments. **D;** Gene expression analysis of wild type (W), non-induced (**-**) and induced (+) cells using Reverse Transcriptase PCR. The products were separated on a 2% agarose gel alongside a 1kb DNA ladder and visualized using ethidium bromide staining. Shown is day 0-48h and day 72h post-RNAi induction. Actin (700bp) was amplified as an internal control alongside NatC catalytic subunit (573bp). The cell densities used for Day 0, 24, 48 and 72h from which RNA was isolated were 1×10^5/ml; 2.73×10^5/ml; 3.75×10^5/ml and 4.7×10^5/ml, respectively.
